# Supplementary material for: Perturbation of Cortical Excitability in a Conditional Model of PCDH19 Disorder
Source: Cells. 2022 Jun 16;11(12):1939. doi: 10.3390/cells11121939 (PMC9222106; doi:10.3390/cells11121939)
Supplement: Supplementary file 1 [file cells-11-01939-s001.zip › cells-1756727-supplementary.pdf]

Supplemental Materials

Material and Methods

Mouse model

The conditional PCDH19 muse was generated by flanking with LoxP sites a 727 bp long region surrounding exon 3 of PCDH19 in mice with a C57BL/6 background (mixed J and N). Mice were genotyped by separation of PCR products (F primer 5′-TCTCCCCCATAGGCTCAACTTTCC- 3′ and R primer 5′-AGTGCCTTTAGGATTCCGAACCACAGG-3′) using agarose gel electrophoresis (yielding a wild-type band of 1053 bp and a floxed one of 1224 bp). Both males and females were used in this study and - since they showed similar activity in the LFP recordings (*Table S1*) - their data were pooled.

We included three experimental groups: 4 males and 7 females injected directly after birth (at postnatal day 1; P1) and recorded in adulthood (around P60), a second group injected at P1 and measured in puberty (around P25), and a third group injected as adults (older than P60) and measured at least 30 days later.

The procedure for viral injections was similar for pups and adults. Pups were anesthetized with isoflurane (Iso-Vet; 5% during induction, 1.5% during the surgery; 200 mL air) delivered through a mouth mask, and adults with a intraperitoneal injection of zoletil-xylazine (50mg/10mg/Kg). Anaesthesia level was assessed using a

tail pinch. Mice were fixed in a stereotactic frame and a small incision was made in the skin. The skull was cleaned with 70% ethanol and then pierced at the target site using a needle in pups or a surgical drill in adults. We targeted an area slightly medial to V1 (2.7 mm posterior to bregma and 2.4 mm lateral to lambda) in the right hemisphere of the mice to prevent causing damage to the recording site. A small glass capillary (40 μm diameter) was slowly lowered into the brain to a depth of 350 μm in pups and 400 μm in adults. Roughly 0.7 μL of virus was injected at a rate of 100 nL/min. After removal of the capillary from the brain, the skull was cleaned again with 70% ethanol and for adults the skin was sutured with a non-absorbable 3-0 filament (Ethicon). Finally, an antiseptic (Betadine) was applied on the skin and the mouse was kept in a warm environment till full recovery.

In vivo local field potential recordings

Animals were prepared for *in vivo* LFP recordings as described previously [22,60]. In brief, the head was fixed in a stereotactic frame, the skin opened and the skull cleaned. A 2-3 mm craniotomy was drilled over the occipital cortex (2.7 mm posterior to bregma and 2.5 mm lateral to lamba) in both hemispheres with the dura mater left intact. A bath containing ACSF was created by surrounding the craniotomy with a thin layer of dental cement. In this way, the cortex could be kept constantly wet. A common reference Ag-AgCl electrode was placed in the bath, while LFPs were recorded with glass micropipettes (impedance ~ 2 MΩ; filled with

ACSF) placed at a depth of 250-300 μm to record from cortical layers II/III using a motorized micromanipulator (Sutter Instrument MPC-200). In the case of γ-aminobutyric acid (GABA; Sigma Aldrich) application, the drug was dissolved in ACSF to a concentration of 5 mM. We then replaced the ACSF in the bath with the ACSF containing GABA, waited for 5 min, recorded for 7.5 min, waited another 5 min and then recorded again for 7.5 min. LFP signals were amplified 1000 times (EXT-02F amplifiers; NPI electronic), band pass filtered (0.1-1000 Hz), cleared from 50 Hz interference (Hum Bug Noise Eliminator; Quest Scientific), and finally oversampled at 10 kHz with 16-bit precision (AD board USB6251; National Instruments).

EEG recordings in behaving mice

Mice were anesthetized with a peritoneal injection of isoflurane (1-4%), fixed in a stereotactic frame, and a cut was made in the skin on top of their skull. The skull was then thoroughly cleaned with a 70% ethanol solution and the head mount glued to the skull. The head mount was further fixed with stainless steel screws placed deep into the skull in contact with the brain surface as in [61]. These screws served as electrodes and were placed in such a way that the 2 EEG channels were placed above the occipital cortex, 1 in each hemisphere, with their corresponding reference electrodes placed more frontally in the same hemisphere. EMG wires were placed in

the trapezius muscles of the mice. The head-mount was further fixed with dental cement and the mice were kept in a warm spot until recovered from the anaesthesia.

The preamplifier provided for band pass filtering of the signal (high pass filter: 0.5-100 Hz for the EEG and 10-200 Hz for the EMG). The data were sampled at 400 Hz and exported in edf format with Sirenia Software 1.7.9 (Pinnacle Technology Inc) for further analyses.

In vivo 2-photon calcium imaging

Time series were acquired at 8.05 Hz (a period of 0.1241 s; 1500 repetitions) at a similar depth of the LFP recordings (200-300 μm) in spiral scan mode. A 20X water immersion objective was used (N20X-PFH-20X Olympus XLUMPLFLN Objective; 1.00 NA) at a resolution of 256*256 pixels and a zoom of 2, leading to an effective resolution of 1.2 μm per pixel and a field of view of about 305*305 μm. Two wavelengths were used: the presence of EGFP was assessed by excitation at 980 nm, while calcium activity was monitored by excitation of the jRGECO1a sensor at 1040 nm.

Molecular Biology and immunohistochemistry

Western Blots were performed on 3 control (injected with an AAV expressing EGFP) and 3 treated (injected with an AAV expressing EGFP-Cre recombinase) mice injected at P1. At the end of the LFP recording, anesthetized mice were sacrificed by

cervical dislocation and a portion of the fluorescent area of the cortex, as well as a portion of cortex from the opposite non-injected hemisphere, was obtained from each mouse. Samples were flash frozen on dry ice and then transferred to -80 °C.

Samples underwent homogenization in modified Radio Immunoprecipitation Assay (RIPA) buffer (50 mM Tris – HCl, 150 mM NaCl, 1 mM EDTA, 1% NP – 40, 1% Triton X100, pH 7.4 and protease inhibitors) and then mixed with Sample Buffer 3X. Homogenates underwent SDS–PAGE and protein were transferred to a 0.2 μm nitrocellulose support (Amersham GE Healthcare) through a wet–tank system (Bio–Rad). Membranes were blocked in 5% skim milk in Tris Buffer Saline (TBS) for 1 hour at room temperature and then washed in TBS with 0,1% Tween 20 detergent (TBST) for 5 minutes at room temperature. Membranes were incubated with primary antibodies prepared in 5% skim milk in TBST 0,1% overnight at 4°C (PCDH19 1:20000, Bethyl Laboratories; GAPDH 1:2000, Santa Cruz Biotechnology). After washing (3 times for 10 min with TBST and twice for 15 min with TBS), membranes were incubated with secondary antibodies for 1 hour at room temperature (1:7500, Li-Cor). Proteins were detected by using the Odyssey CLx detector system and quantified by Image Studio software program.

Combined in situ hybridization (ISH) and immunohistochemistry (IHC) analysis for PCDH19 exon 1 and EGFP was performed on floxed (mosaic), and non-floxed (control) adult mice injected at P1 with EGFP-Cre recombinase as previously described [62]. Briefly, deeply anesthetized mice were sacrificed at the end of the

experiment and their brains quickly dissected, embedded in TissueTek (Sakura) and frozen at 80 °C. Fourteen-micrometre cryosections were cut on the coronal plane incubated overnight at 4 °C with primary mouse anti-EGFP antibody (1:2000, Thermo Fisher Scientific Inc.), followed by a 2-hour incubation at room temperature with an Alexa Fluor 488 goat anti-rabbit antibody (1:500, Thermo Fisher Scientific Inc.). Following IHC, ISH was performed using a fluorescein-labeled *Pcdh19* antisense riboprobe (0.9 Kb). A chromogenic reaction using NBT/BCIP substrate solution (Roche) was performed to visualize the dig-labelled riboprobe. Images were acquired with a Nikon SMZ18 (low magnification images; SHR plan apo 1x objective; Figure S1D left) and a Zeiss Airyscan (20x water immersion objective; Figure S1D right).

Immunohistochemistry for Cre recombinase was performed on the brains of most of the recorded animals injected at P1. At the end of the LFP recording, deeply anesthetized mice were transcardially perfused with 4% paraformaldehyde without regaining consciousness. Coronal brain sections (60 μm thick) were cut using a vibratome and rinsed 3 times for 15 min with PBS. Blocking was performed for 1 hr at room temperature (blocking solution: 3% BSA; 0.4% Triton-X100; in PBS). Sections were incubated with primary antibody in blocking solution (mouse anti-Cre; 1:100; Sigma Aldrich) overnight at 4 °C, rinsed 3 times in PBS, and incubated with secondary antibody (anti-mouse TRITC; 1:300; Sigma Aldrich) for 1 hr at room

temperature. After 3 additional rinsing steps in PBS, slices were mounted in Fluoroshield™ with DAPI (Sigma Aldrich) to stain nuclei.

Data analyses: electrophysiology

LFP traces were band-pass filtered with zero phase distortion. Signal strength in various frequency bands was calculated by computing the root mean square (RMS) of the filtered signal. The frequency-time analysis was calculated by computing the RMS of the filtered signal in a moving 0.25 s window with 0.05 s overlap. Up states were detected by analysis of the distribution of the logarithm of signal strength in the β-σ (9-25 Hz) band over time. This distribution was bimodal, in correspondence with up states (high β signal strength) and down states (low β signal strength). The distribution was fitted with two Gaussian curves, and the threshold was set at the intersection of the two distributions. The automated segmentation of the LFP data allowed the non-assisted computation of all metrics relative to SWA.

Up state slopes were calculated based on [23,24]. In brief, we computed the LFP filtered in the δ (0.5-4 Hz) range between the start and end time of each detected up state. The average slope was calculated by dividing the amplitude of the most prominent negative peak by the interval elapsing from either the previous zero crossing (first segment of the up state) or the subsequent zero crossing (second segment of the up state).

Units were extracted from the data as suggested by [63]. Briefly, we high-pass filtered the LFP at 300 Hz as described above. We then automatically set a threshold according to

$$Thr = 4 * median\left\{\frac{|x|}{0.6745}\right\}$$

where x is the filtered LFP. Spikes crossing the threshold were labelled as units and their frequency and correlation with up state timing was determined.

The slope of the power spectrum was computed according to an algorithm developed by [64] (https://github.com/DidiLamers/PCDH19_FOOOF). First, we calculated the power spectrum of each LFP trace using a multitaper method (*mtspectrumc* function of the Chronux Toolbox; [65]). These power spectra were then fitted with an aperiodic signal ($1/f$) and periodic oscillatory components based on Gaussian model fits. The aperiodic exponential function

$$L = b - \log{(k + F^X)}$$

where F represents the input frequencies, is characterized by an offset, b, a 'knee' parameter, k (which, if not equal to zero, adds a bend to the aperiodic signal), and the exponent or slope, X. We chose to fit the power spectrum with $k = 0$ between 9-25 Hz as this gave a good fit with low error and high R squared values. We averaged the exponents and error values of all LFP traces for each animal.

Finally, we quantified bursts of hyperexcitable activity by the automated detection of episodes of high β-σ (9-25 Hz) signal strength as for up state detection, but with the

added restraint that the episode should be at least 4 times as long as an average up state of control animals (4*0.85 s). In this way, we ensured that the labelled event was not a physiologically correct up state. EEG signals were evaluated for the presence of β oscillations in the same way. Hypersynchronous peaks were automatically detected as episodes of high γ (40-100 Hz) signal strength (again as described for up state detection) of a duration between 30 and 300 ms, with a downward slope of at least 10 mV/s (calculated from the peak till the half maximum of the peak) and an amplitude of at least 0.3 mV. Timing of these events were correlated with timing of up states in the opposite, control hemisphere. Values were averaged per animal.

The EEG data from the behaving mice were used to score sleep phases by an adaptation of the algorithm termed SCOPRISM [66] and our code is available at https://github.com/DidiLamers/PCDH19_sleepscoring.

In brief, epochs of 4 seconds were scored based on the RMS of the EMG signal to distinguish wake from sleep. The identification of REM and NREM sleep segments was performed by computing the ratio of the EEG spectral power ratio in the θ (6-9 Hz) to the δ (0.5-4 Hz) band: NREM (slow wave sleep) is characterized by high δ power and relatively lower θ power. Scoring was then refined based on scoring results of surrounding epochs. Data were cleared by artefacts by ignoring all epochs with an EEG or EMG value above a manually set threshold (usually set to 300 μV).

Data analyses: 2 photon imaging

In brief, images were binned 2*2 and the time series representing the fluorescence fluctuation for each pixel was computed as in [67]

$$\Delta F(x,y,t)/F_0 = \frac{f(x,y,t) - \langle f(x,y)\rangle}{\langle f(x,y)\rangle}$$

with $f(x,y,t)$ representing the fluorescence signal of that pixel and $\langle f(x,y)\rangle$ the median fluorescence. We then used the statistics of the $\Delta F(x,y,t)/F_0$ signal to create a binarized image. In the absence of any calcium activity, $\Delta F(x,y,t)/F_0$ will be normally distributed. Ca^{2+} activity appears as a tail on the right side of the distribution. We computed the threshold at 2 times the standard deviation of the Gaussian distribution of the $\Delta F(x,y,t)/F_0$ process and pixels of each frame were labelled as either '0' when below threshold or '1' if over threshold. In this way, the binarized stack contains only physiologically relevant events. We added a further restraint by rejecting all isolated pixels.

The binary file was saved and imported in ImageJ, where we set ROIs representing 'green' (expressing EGFP-Cre recombinase and jRGECO1a) or 'red' (expressing jRGECO1a only) cells based on the summed fluorescence signal in the red and green channels. We then counted calcium transients using ImageJ's *Analyze Particles* macro on the binary file in the entire field of view and the 'red' and 'green' cells. These data were used to calculate the transient frequency as well as the percentage of calcium transients occurring during up states in each cell and field of view.

Data analyses: statistics

All statistical analyses were performed in GraphPad Prism 5 software (GraphPad Inc., San Diego, CA). All statistical hypotheses were tested with non-parametric tests. If not otherwise specified, we used a Wilcoxon signed-rank test for paired measurements (i.e. to compare the two hemispheres) and a Mann Whitney test to compare two experimental groups.

Supplementary Figures

Figure S1

Generation of the PCDH19 mosaic model

All experiments were performed on a conditional mouse line where exon 3 of PCDH19 is flanked by LoxP sequences (figure S1A). We induced a patch of Cre-mediated recombination by a focal injection of an AAV expressing EGFP-Cre. To confirm that PCDH19 expression was reduced, we resorted to a quantitative Western Blot (WB). We found that PCDH19 protein expression is reduced in the injected hemisphere compared to that of EGFP injected control mice (figure S1B; P=0.003). This demonstrates that, even though Cre removes only exon 3, the entire protein is missing. It is likely that excision of exon 3 causes a frameshift and premature stop codon in the gene upon which the mRNA is degraded by nonsense-

mediated decay. Immunohistochemistry (IHC) for Cre-recombinase shows that at the injection site we have obtained a 'salt-and-pepper' like mosaicism since not all neurons expresses Cre (Figure S1C). Further evidence for this notion is provided by an In Situ Hybridization (ISH) for exon 1 of PCDH19 showing that the EGFP-Cre recombinase injection strongly reduces the staining in floxed mice, but not in non-floxed littermates (Figure S1D). We therefore conclude that upon Cre-mediated recombination of exon 3, mRNA expression of exon 1 is also reduced. The ISH confirms previous studies demonstrating that PCDH19 is mainly expressed in layers II/III and layer V of the cortex [8,12,68]. Within these cortical layers PCDH19 is not present in every cell [69]; Figure S1D bottom right). We therefore conclude that Cre recombinase injection causes a cellular mosaicism.

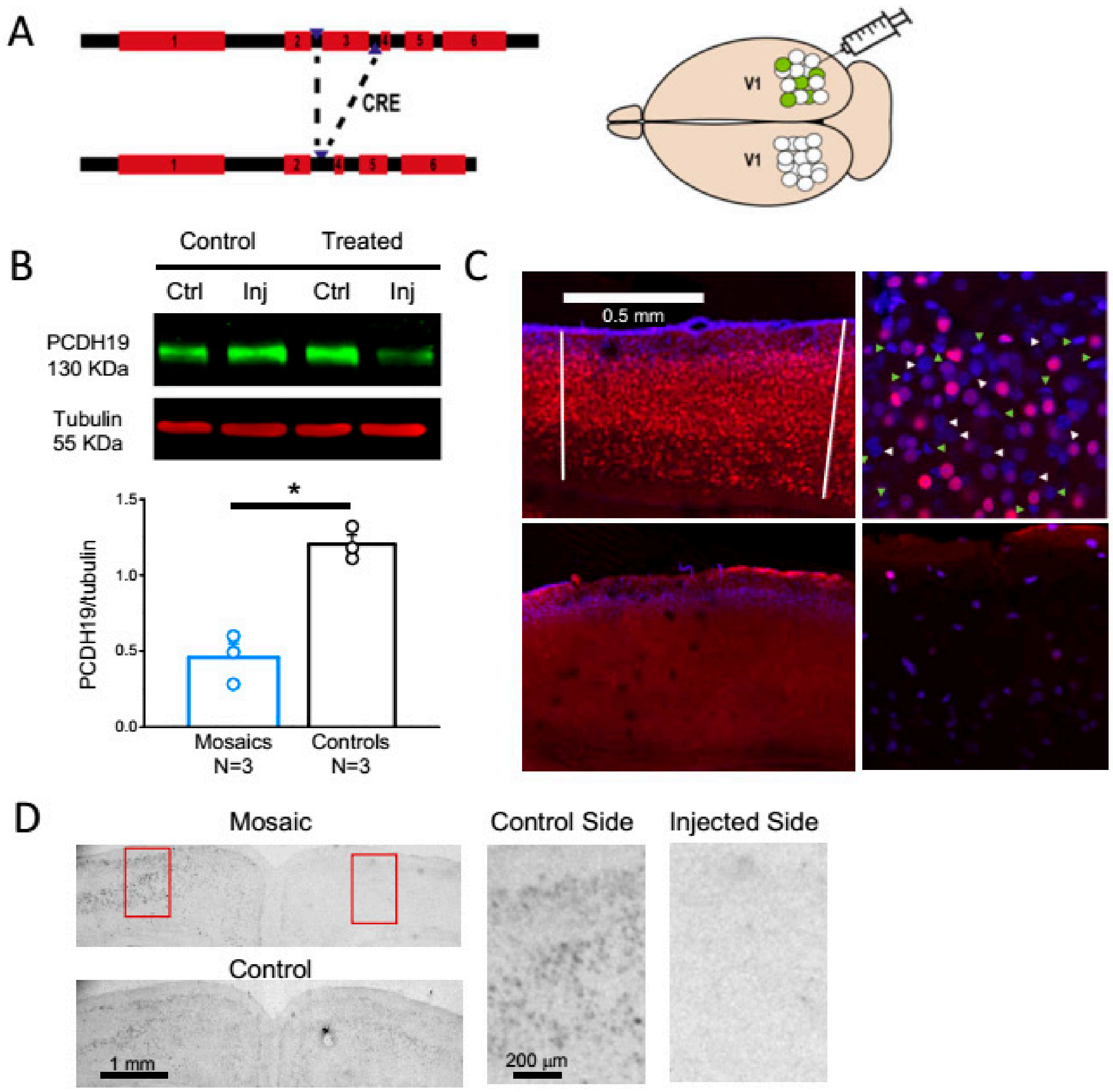

Figure S1: Focal model of mosaic PCDH19 expression. A) Creation of the mouse model. Exon 3 of the PCDH19 gene was placed between LoxP sites, allowing for excision of the exon upon introduction of Cre recombinase. **B**) Quantitative WB confirms a reduction in PCDH19 protein expression in the hemisphere injected with the Cre vector (in blue) versus control (floxed mice injected with the EGFP vector, in black) by about half (average PCDH19/tubulin value of injected hemisphere/control hemisphere of 0.46 ± 0.09 in 'Mosaics' versus 1.20 ± 0.06 for controls, * P<0.005 t-test.

Top image shows the WB for two example animals: one control and one mosaic. Staining for PCDH19 (in green) was normalized by the expression of housekeeping gene Tubulin (in red) and values for the injected hemisphere (inj) were normalized by values for the control (ctrl) hemisphere. **C**) IHC of Cre recombinase (in red) in the injected (top) and control (bottom) hemisphere of an EGFP-Cre recombinase injected floxed mouse. Cre positive (green arrows) and negative (white arrows) cells are visible in the higher magnification image (on the right) in the injected hemisphere, with no Cre present in the control hemisphere. The white lines indicate the edges of the primary visual cortex. Section located 3.5 mm posterior to bregma. **D**) ISH for PCDH19 exon 1 of an injected floxed littermate (top) and an injected non-floxed mouse (bottom). The expression of exon 1 is strongly reduced in the cortex of the injected hemisphere of the floxed, but not of the control mouse. The rectangles are drawn in correspondence of the magnified insets on the right. Section located 2.5 mm posterior to bregma. All mice in this figure were injected at P1 and sacrificed as adults.

Figure S2.

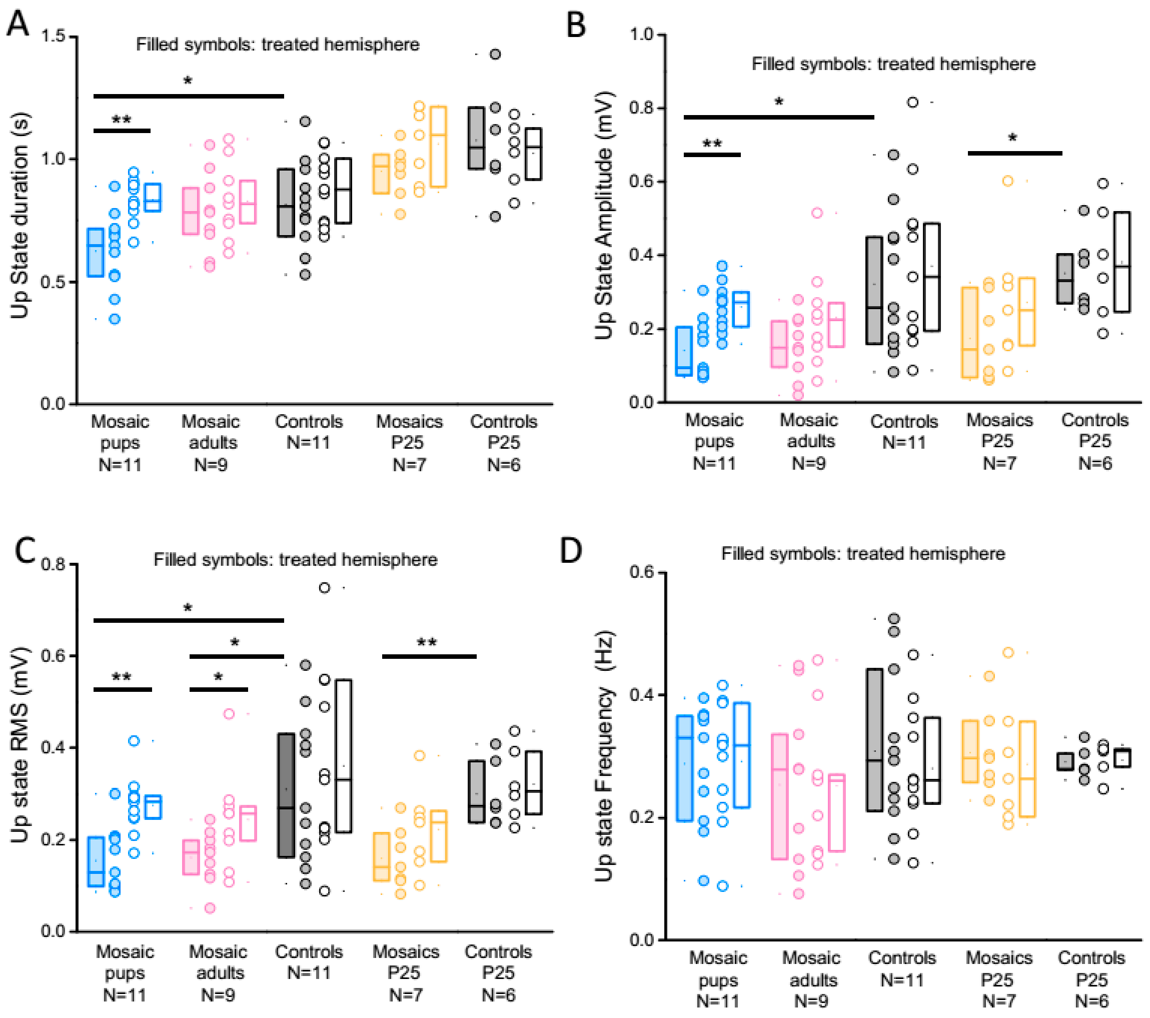

Figure S2: Up state characteristics are affected by PCDH19 mosaicism. Up state duration **A**), amplitude **B**), and RMS strength **C**) are all reduced in PCDH19 mosaic tissue, while their frequency **D**) is not altered. All graphs represent box plots with whiskers of minimum and maximum values of all three experimental groups, while dots represent the average value per animal with the values of the two hemispheres connected by lines. Significant differences are indicated with asterisks (** P<0.005, *

P<0.05; Wilcoxon signed-rank test for paired measurements; Mann Whitney test for non-paired). US duration is significantly reduced in the injected hemisphere of Mosaic pups compared to their control hemisphere (P<0.001) and to the injected hemisphere of Control animals (P<0.03). US amplitude is similarly reduced in the injected hemisphere of Mosaic pups (P<0.001 compared to the control hemisphere and P<0.02 compared to the injected hemisphere of Controls). Also, the injected hemisphere of mice recorded at P25 displays a reduced US amplitude, though only compared to the injected hemisphere of Control animals (P<0.04). RMS signal strength of up states is reduced in Mosaic pups (injected hemisphere versus control hemisphere: P<0.001, injected hemisphere of Mosaics versus injected hemisphere of Controls: P<0.02), Mosaic Adults (injected versus control hemisphere: P<0.02; injected hemisphere of Mosaic adults versus that of Controls: P<0.05) and mice recorded at P25 (injected hemisphere of Mosaic P25 animals versus that of Controls: P<0.005). US frequency is unaltered in all cases.

Figure S3.

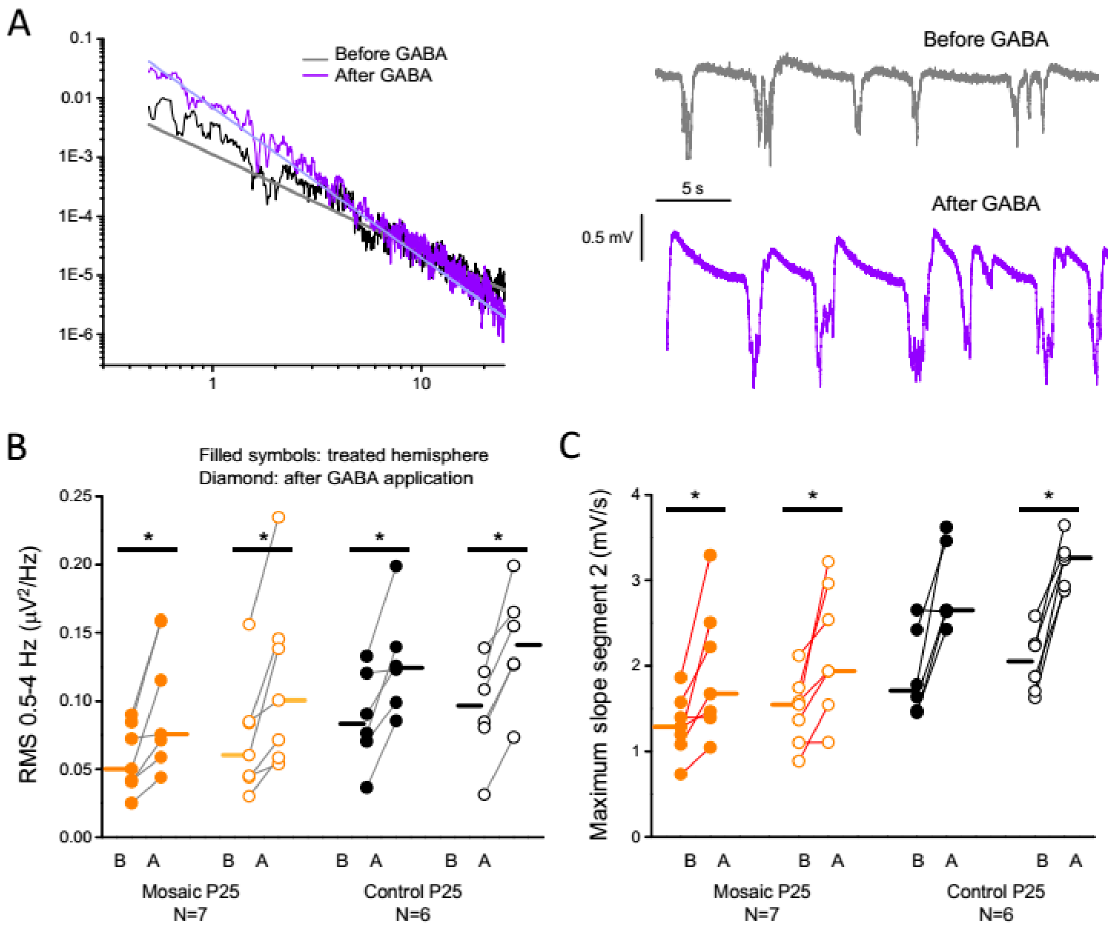

Figure S3: PCDH19 mosaic animals have an increased excitation to inhibition ratio. **A**) Example power spectrum of a control animal recorded at P25 (black line) and the exponential fit (thick line) computed with the FOOF algorithm. After GABA application, the power spectra changed as shown by the magenta line and its exponential fit (thick magenta line). This spectrum is steeper (an exponent of 2.54 versus 1.64 before GABA application). The Root Mean Square Error of both fits is 0.12 and the R^2 is 0.95 (before GABA) and 0.97 (after GABA). The corresponding LFP traces are shown to the right (before GABA application in grey, after in magenta).

Note the large amplitude slow waves after GABA application. **B**) Bar plots representing median RMS strength in the 0.5-4 Hz range (left) and the US slope (right). Dot plots display the values of each animal, with lines connecting the values before (B) and after (A) GABA application. In red are the mosaic animals recorded at P25, in black their controls. Open dots represent the injected hemisphere, closed ones the control hemisphere. GABA application significantly increases SWA (the RMS in the 0.5-4 Hz range; before versus after GABA application of the injected hemisphere of Mosaics $P=0.02$, their control hemisphere $P<0.02$, the injected hemisphere of Controls $P<0.03$, and the control hemisphere of Controls: $P<0.03$) as well as the slope of USs **(C)** (before versus after GABA application of the injected hemisphere of Mosaics $P<0.03$, their control hemisphere $P<0.02$, the injected hemisphere of Controls $P<0.06$, and the control hemisphere of Controls: $P<0.03$).

Table S1.

Since PCDH19 Epilepsy primarily affects females, we tested whether there was a difference in phenotype between male and female mice. In agreement with the hypothesis that a mosaic PCDH19 expression causes the disease, we found that male and female mice showed a similar reduction in δ oscillatory strength (figure 3B; inset). Since we recorded only four males, the difference was not significant for males (P=0.1) but it was for females (P=0.02). Note that none of the analyses that we performed on the LFP data showed differences between males and females in the 'Mosaic Pups' experimental group. In all the upcoming graphs the values of male and female mice were therefore pooled.

Table S1: LFP characteristics of 'Mosaic Pups' in males versus females. All values are mean with SD. Males do not show significant differences compared to females and the two experimental groups were therefore pooled.

	Male (N=4) treated hemisphere	*Male (N=4) control hemisphere*	*Female (N=7) treated hemisphere*	*Female (N=7) control hemisphere*
RMS 0.5-4 Hz (mV)	0.045 ± 0.010	0.092 ± 0.030	0.046 ± 0.032	0.088 ± 0.032
Up State Duration (s)	0.69 ± 0.04	0.83 ± 0.04	0.59 ± 0.19	0.83 ± 0.11
Up State Amplitude (mV)	0.16 ± 0.06	0.28 ± 0.02	0.13 ± 0.08	0.25 ± 0.08
Up State RMS (mV)	0.18 ± 0.052	0.28 ± 0.034	0.14 ± 0.077	0.28 ± 0.08
Up State Frequency (Hz)	0.33 ± 0.092	0.34 ± 0.088	0.26 ± 0.101	0.27 ± 0.101
Maximum Up State slope segment 2 (mV/s)	0.95 ± 0.34	2.08 ± 0.70	0.99 ± 0.66	2.22 ± 0.82
Unit Frequency (Hz)	1.06 ± 0.24	5.90 ± 3.02	1.36 ± 0.48	3.30 ± 1.80
Units in Up State (%)	50.4 ± 9.8	88.2 ± 8.0	35.9 ± 27.3	61.5 ± 22.0
Slope Power Spectrum (exponent)	1.58 ± 0.06	1.90 ± 0.08	1.53 ± 0.37	2.07 ± 0.29

Table S2: Up State slopes are reduced in mosaic mice. All values are mean (mV/s) ± SEM.

	Average slope segment 1	*Maximum slope segment 1*	*Average slope segment 2*	*Maximum slope segment 2*
'Mosaic Pups' treated hemisphere	-0.660 ± 0.098	-1.047 ± 0.164	0.595 ± 0.092	0.973 ± 0.165
'Mosaic Pups' control hemisphere	-1.481 ± 0.150	-2.400 ± 0.227	1.298 ± 0.139	2.169 ± 0.222
'Mosaic Adults' treated hemisphere	-0.723 ± 0.088	-1.218 ± 0.164	0.777 ± 0.098	1.296 ± 0.175
'Mosaic Adults' control hemisphere	-1.190 ± 0.154	-2.065 ± 0.252	1.180 ± 0.178	1.977 ± 0.299
'Controls' treated hemisphere	-1.623 ± 0.242	-2.672 ± 0.411	1.340 ± 0.169	2.350 ± 0.305
'Controls' control hemisphere	-1.963 ± 0.301	-3.194 ± 0.494	1.500 ± 0.193	2.773 ± 0.394
'Mosaic P25' treated hemisphere	-0.842 ± 0.078	-1.405 ± 0.145	0.806 ± 0.069	1.304 ± 0.136
'Mosaic P25' control hemisphere	-0.898 ± 0.090	-1.569 ± 0.180	0.844 ± 0.065	1.479 ± 0.155
'Controls P25' treated hemisphere	-1.076 ± 0.118	-1.850 ± 0.209	1.100 ± 0.129	1.904 ± 0.208
'Controls P25' control hemisphere	-1.135 ± 0.048	-1.992 ± 0.121	1.168 ± 0.089	2.047 ± 0.151

Table S3: Root Mean Square Error and R^2 of the fit of the power spectra in the 0.5-25 Hz range of our mice with an exponential function (Donoghue *et al.*, 2020). All values show mean ± SEM of all animals in each experimental group.

	RMSE	*R^2*
'Mosaic Pups' treated hemisphere	0.132 ± 0.005	0.914 ± 0.009
'Mosaic Pups' control hemisphere	0.125 ± 0.003	0.953 ± 0.003
'Mosaic Adults' treated hemisphere	0.135 ± 0.004	0.922 ± 0.015
'Mosaic Adults' control hemisphere	0.125 ± 0.003	0.940 ± 0.006
'Controls' treated hemisphere	0.126 ± 0.003	0.951 ± 0.006
'Controls' control hemisphere	0.127 ± 0.004	0.955 ± 0.006
'Mosaic P25' treated hemisphere	0.121 ± 0.004	0.940 ± 0.005
'Mosaic P25' control hemisphere	0.123 ± 0.002	0.944 ± 0.004
'Controls P25' treated hemisphere	0.140 ± 0.011	0.942 ± 0.012
'Controls P25' control hemisphere	0.140 ± 0.010	0.944 ± 0.006

References

60. Trovato, F.; Parra, R.; Pracucci, E.; Landi, S.; Cozzolino, O.; Nardi, G.; Cruciani, F.; Mosti, L.; Cwetsch, A.; Cancedda, L. et al. A Cre-amplifier to generate and detect genetic mosaics in vivo. *bioRxiv*, **2019**. 715490. https://doi.org/10.1101/715490
61. Mazziotti, R.; Cacciante, F.; Sagona, G.; Lupori, L.; Gennaro, M.; Putignano, E.; Alessandrì, M.G.; Ferrari, A.; Battini, R.; Cioni, G.; et al. Novel translational phenotypes and biomarkers for creatine transporter deficiency. *Brain Commun.* **2020**, *2*, https://doi.org/10.1093/braincomms/fcaa089.
62. Napolitano, F.; De Rosa, A.; Russo, R.; Di Maio, A.; Garofalo, M.; Federici, M.; Migliarini, S.; LeDonne, A.; Rizzo, F.R.; Avallone, L.; et al. The striatal-enriched protein Rhes is a critical modulator of cocaine-induced molecular and behavioral responses. *Sci. Rep.* **2019**, *9*, 1–12, https://doi.org/10.1038/s41598-019-51839-w.
63. Quiroga R.Q.; Nadasdy Z.; Ben-Shaul Y. Unsupervised spike detection and sorting with wavelets and superparamagnetic clustering. *Neural. Comput.* **2004**, *16*: 1661–1687.

64. Donoghue, T.; Haller, M.; Peterson, E.J.; Varma, P.; Sebastian, P.; Gao, R.; Noto, T.; Lara, A.H.; Wallis, J.D.; Knight, R.T.; et al. Parameterizing neural power spectra into periodic and aperiodic components. *Nat. Neurosci.* **2020**, *23*, 1655–1665, https://doi.org/10.1038/s41593-020-00744-x.
65. Bokil H.; Andrews P.; Kulkarni J.E.; Mehta S.; Mitra P. Chronux: A Platform for Analyzing Neural Signals. *J. Neurosci. Methods* **2010**, *192,* 146–151.
66. Bastianini, S.; Berteotti, C.; Gabrielli, A.; Del Vecchio, F.; Amici, R.; Alexandre, C.; Scammell, T.E.; Gazea, M.; Kimura, M.; Martire, V.L.; et al. SCOPRISM: A new algorithm for automatic sleep scoring in mice. *J. Neurosci. Methods* **2014**, *235*, 277–284, https://doi.org/10.1016/j.jneumeth.2014.07.018.
67. Cozzolino O.; Sicca F.; Paoli E.; Trovato F.; Santorelli F.M.; Ratto G.M., Marchese M. Evolution of Epileptiform Activity in Zebrafish by Statistical-Based Integration of Electrophysiology and 2-Photon Ca 2^+ Imaging. *Cells* **2020**, *9*, 769.
68. Pederick, D.; Homan, C.; Jaehne, E.; Piltz, S.G.; Haines, B.P.; Baune, B.T.; Jolly, L.; Hughes, J.N.; Gecz, J.; Thomas, P.Q. Pcdh19 Loss-of-Function Increases Neuronal Migration In Vitro but is Dispensable for Brain Development in Mice. *Sci. Rep.* **2016**, *6*, 26765, https://doi.org/10.1038/srep26765.
69. Krishna-K K.; Hertel N.; Redies C. Cadherin expression in the somatosensory cortex: evidence for a combinatorial molecular code at the single-cell level. *Neuroscience* **2011**, *175*, 37–48.
